# Continual Radar Intra-Pulse Modulation Recognition via Multiview Replay and Class-Balanced Classifier Fine-Tuning

**DOI:** 10.3390/s26175494

**Published:** 2026-08-30

**Authors:** Yilin Liu, Xiaofang Wu, Caoyi Mei, Shuo Yu

**Affiliations:** Institute of Systems Engineering, Academy of Military Sciences of the People’s Liberation Army, Beijing 100191, China; liuyilin123@hrbeu.edu.cn (Y.L.); meicaoyi20@alumni.nudt.edu.cn (C.M.); yushuo@alumni.nudt.edu.cn (S.Y.)

**Keywords:** radar signal recognition, class-incremental learning, classifier fine-tuning, multiview fusion, continual sensing, intra-pulse modulation

## Abstract

Continually deployed radar recognizers must acquire emerging intra-pulse modulation classes without erasing learned signal types under small replay memories. We present MM-CILNet, a radar-specific multiview replay framework that combines complementary I/Q and short-time Fourier transform (STFT) representations, sample-wise fusion, class-balanced memory, and a one-epoch update of three classifier heads after each incremental session. On the 20-class CIL-20 protocol with 12 matched seeds, MM-CILNet improves final-session All and Old accuracy by 2.7 percentage points each over matched Replay (All: p=0.0034, Cohen’s d=1.07; Old: p=0.0103, d=0.89). Under the shared configuration, it achieves the best All and Old accuracy among the listed radar-adapted CIL methods and the lowest average forgetting among the four methods with recoverable session histories. Equal-budget controls identify significant post-session effects on All and Old accuracy and show that trainable scope and sampling determine the retention–plasticity operating point. The Old-accuracy advantage persists at the tested 400- and 800-sample budgets and across five class orders. Under 2.5:1 recent-class-heavy replay, Old accuracy decreases by 1.63–2.01 pp and forgetting increases by 3.66–4.69 pp relative to class-balanced replay. These results establish class-balanced memory as the retention-oriented operating choice and provide an integrated protocol for memory-constrained continual radar recognition.

## 1. Introduction

Radar intra-pulse modulation recognition is a core sensing task in electronic reconnaissance, spectrum monitoring, and cognitive electronic warfare. Modern radar sensors operate in increasingly congested and dynamic electromagnetic environments where new waveform classes appear incrementally after initial deployment [1,2]. A practical recognition system must therefore acquire new modulation types, while retaining previously learned ones, without storing all historical samples or retraining from scratch. This requirement defines a class-incremental learning (CIL) problem under real-world sensing constraints: limited memory, evolving emitter populations, and a wide signal-to-noise-ratio (SNR) range.

The CIL problem has been extensively studied in computer vision [3,4,5,6,7,8], where the catastrophic forgetting of old classes is the central challenge. Radar signal recognition adds several practical sensing considerations that distinguish it from vision-domain CIL. First, raw radar signals are time-series I/Q complex samples, not images; their discriminative information resides in instantaneous phase continuity, frequency trajectories, and intra-pulse modulation structure. Second, the CIL-20 protocol evaluates a wide SNR range, from −18 dB to +12 dB, over which the usefulness of different signal representations varies with channel conditions. Third, modern radar waveforms increasingly use compound modulation types that combine multiple coding schemes within a single pulse, creating intra-class variability that challenges fixed fusion strategies.

Prior work on radar signal recognition has mainly considered closed-set classification [9,10,11,12], where all classes are known during training. Radar systems can exploit complementary information from either different sensors or different representations of the same measurement. Multimodal ISAC systems, for example, integrate camera and mmWave radar observations for V2I tracking [13]. Multiview modulation recognition follows the latter approach by constructing I/Q and time-frequency inputs from the same received signal [14,15,16]. However, existing multiview studies assume a fixed class set, leaving open whether these complementary representations remain beneficial when a model must learn new classes without forgetting those learned earlier.

This paper makes four contributions:

- We formulate continual radar intra-pulse modulation recognition and present MM-CILNet, a multiview replay framework that integrates I/Q and STFT representations, class-balanced replay, post-session classifier-head fine-tuning, and sample-wise gated fusion.
- On the 20-class CIL-20 protocol, MM-CILNet improves final-session All and Old accuracy over matched Replay by 2.73 and 2.67 percentage points, respectively. Under the shared configuration, it achieves the best All and Old accuracy among the listed radar-adapted CIL methods and the lowest average forgetting among the four methods with recoverable session histories.
- Through a 12-seed, equal-budget decomposition, we isolate fusion, post-stage parameter scope, and sampling. The factorial analysis identifies a general post-session effect on All and Old accuracy and shows how trainable scope and sample allocation select different retention–plasticity operating points.
- We evaluate the complete protocol across memory budgets, class orders, replay allocations, distribution shifts, and three related datasets. The Old-accuracy advantage persists at the tested 400- and 800-sample budgets and across all five tested class orders; under 2.5:1 recent-class-heavy replay, the Old-accuracy and forgetting endpoints identify class-balanced memory as the retention-oriented choice.

## 2. Related Work

### 2.1. Radar Signal Recognition

Radar signal recognition has evolved from handcrafted feature engineering to deep learning. Early work used time-frequency transforms with expert-defined features [1]. Convolutional networks applied to time-frequency images [9,10] and directly to I/Q sequences [17,18] improved closed-set accuracy. Sequence models have also provided a unified treatment of radar and communications signals together with a public classification dataset [19]. Recent fixed-set studies address limited labels through semi-supervised time-frequency recognition [20] and variable noise through the AIMC-Spec intrapulse benchmark [21]. Adjacent radar-recognition work addresses scarce working-mode sequences through generative augmentation [22] and low-SNR jamming recognition through time-, frequency-, and time-frequency-derived feature fusion [23]. Multiview approaches combining multiple representations [14,15,16] further improved robustness. These studies strengthen recognition under fixed label vocabularies or adjacent radar-signal tasks. MM-CILNet instead targets sequential expansion of intrapulse modulation classes and identifies the components that preserve previously learned classes during that expansion.

### 2.2. Class-Incremental Learning

Class-incremental learning addresses catastrophic forgetting when models are sequentially updated with new classes. Foundational regularization methods stabilize parameters important to earlier tasks [24], while output distillation preserves previous predictions without retaining the original training set [25]. Contemporary CIL families include replay-based methods [3,6], bias-correction methods [4,7], distillation-based methods [5], and feature-boosting methods [8]. Replay stores a subset of old-class samples and interleaves them with new-class data during incremental training. Recent replay research improves exemplar selection using distribution and decision-boundary criteria [26], adapts coreset construction to time-varying class frequencies [27], and jointly optimizes exemplars and knowledge transfer through data and feature distillation [28]. Bias correction addresses the classifier tendency to favor recently seen classes by post-hoc adjustment of output logits or classifier weights, whereas distillation penalizes changes in feature representations or output distributions. Our work builds on replay and decomposes the contributions of dual-representation fusion, post-session optimization, parameter scope, and sampling.

### 2.3. Multiview Learning for Radar Signals

Radar signals can be represented in multiple domains. I/Q complex samples preserve instantaneous phase and amplitude, while time-frequency representations (STFT, WVD) expose modulation structure. General gated multimodal units learn intermediate representations by controlling the contribution of modality-specific features [29]. Radar-specific work has shown benefits from fusing multiple representations in closed-set classification [14,15,16,30,31]. Common strategies include early concatenation, intermediate attention, and late averaging. Our experiments extend this comparison to CIL and test representation and post-stage effects in the same matched-seed design.

### 2.4. Classifier Rebalancing in Class-Incremental Learning

Classifier rebalancing has been studied in general CIL. BiC [4] learns a bias-correction layer on class-balanced validation data after each session. WA [7] aligns old and new classifier weight norms to reduce prediction bias toward recently seen classes. LUCIR [32] combines cosine normalization with margin-based constraints for a more balanced feature space. MM-CILNet instead fine-tunes its three existing cosine classifier heads with cross-entropy on updated replay memory while freezing both encoders and the gate. We call this operation class-balanced classifier-head fine-tuning: it rebalances decision boundaries but does not calibrate predictive probabilities.

Classifier-level CIL architectures have also combined probability dampening with cascaded gates that rescale task-wise classifier outputs [33]. Those gates modify the incremental classifier, whereas the MM-CILNet sample-wise gate mixes the I/Q and STFT embeddings before its shared classifier. This distinction separates classifier-output intervention from representation fusion.

Methodologically, MM-CILNet joins same-pulse multiview learning and class-incremental replay at the point where their complementary strengths matter. Fixed-set multiview methods fuse signal representations without addressing sequential class expansion, whereas generic replay and classifier-rebalancing methods do not exploit the relationship between I/Q and STFT views of one radar pulse. MM-CILNet couples two representation-specific encoders and auxiliary heads with sample-wise fusion, class-balanced replay, and a bounded post-session head update. Its contribution is this radar-specific continual-recognition protocol together with a matched decomposition that identifies how representation, post-stage scope, and sampling govern retention and plasticity.

Table 1 makes the method-level distinction explicit. The closest families each address only part of the problem: fixed-set radar multiview methods learn complementary representations without class expansion, and established CIL methods manage class expansion without a same-pulse I/Q–STFT fusion interface. MM-CILNet couples both in one controlled protocol and evaluates the coupling through matched factor-level interventions.

## 3. Methods

### 3.1. Problem Formulation

**Figure 1:**
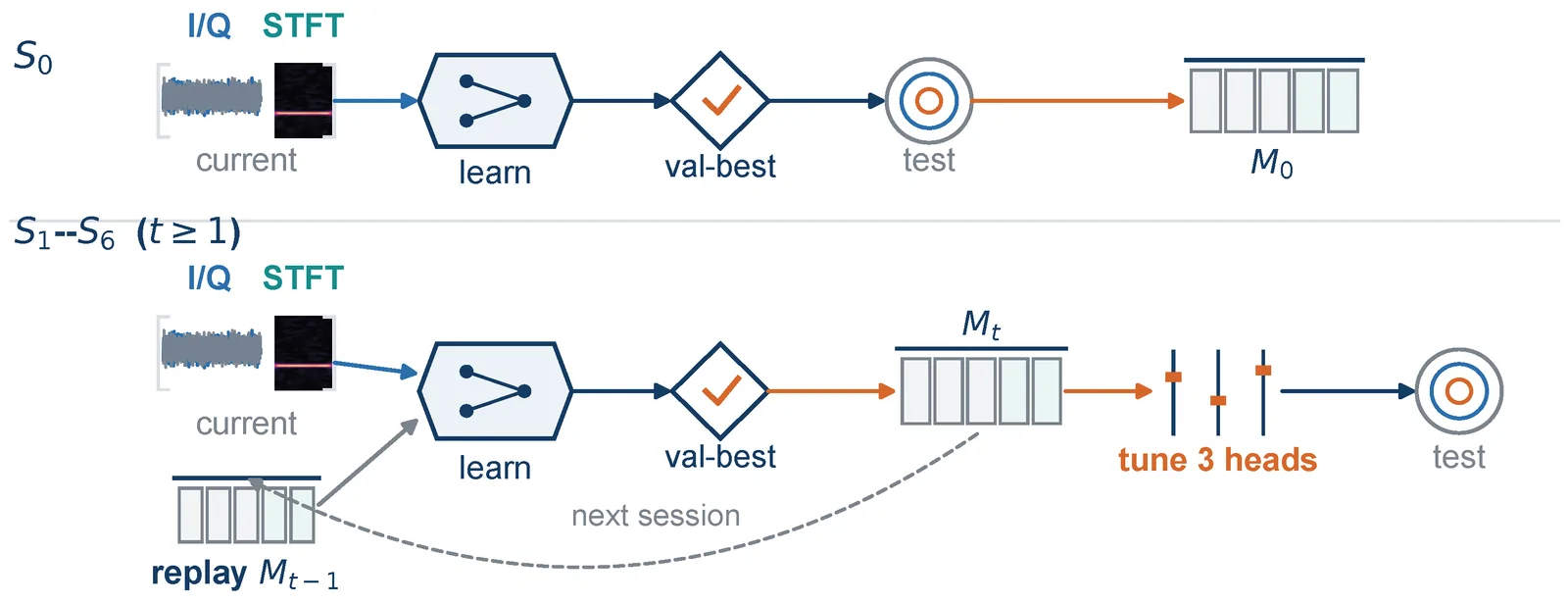
Base and incremental lifecycles of MM-CILNet. I/Q and STFT are deterministic views of the same pulse. At $S_{0}$, the validation-selected checkpoint is restored and tested before $M_{0}$ is initialized; at t≥1, current-plus-replay training is followed by checkpoint restoration, memory update, three-head fine-tuning, and test evaluation.

Figure 1 provides an overview of the proposed framework. Let $D_{t}={\left\{\left(x_{i}^{\mathrm{IQ}},x_{i}^{\mathrm{TF}},y_{i}\right)\right\}}_{i=1}^{N_{t}}$ denote the training set at session *t*, where $x_{i}^{\mathrm{IQ}}\in C^{T}$ is the complex I/Q sequence of length T=1024, $x_{i}^{\mathrm{TF}}\in R^{H\times W}$ is the STFT magnitude image of size 224×224, and $y_{i}\in C_{t}$ is the modulation class label. The class set $C_{t}$ expands as $C_{t}=C_{t-1}\cup C_{t}^{\mathrm{new}}$, with $C_{t-1}\cap C_{t}^{\mathrm{new}}=\emptyset$. A fixed-size replay memory M stores a subset of old-class samples, updated to remain class-balanced across all seen classes. The goal is to learn a classifier $f_{t}:X^{\mathrm{IQ}}\times X^{\mathrm{TF}}\to C_{t}$ that performs well on all classes observed up to session *t*, without access to raw samples from sessions 0 through t−1 beyond those in M.

### 3.2. Dual-Representation Feature Extraction

**Figure 2:**
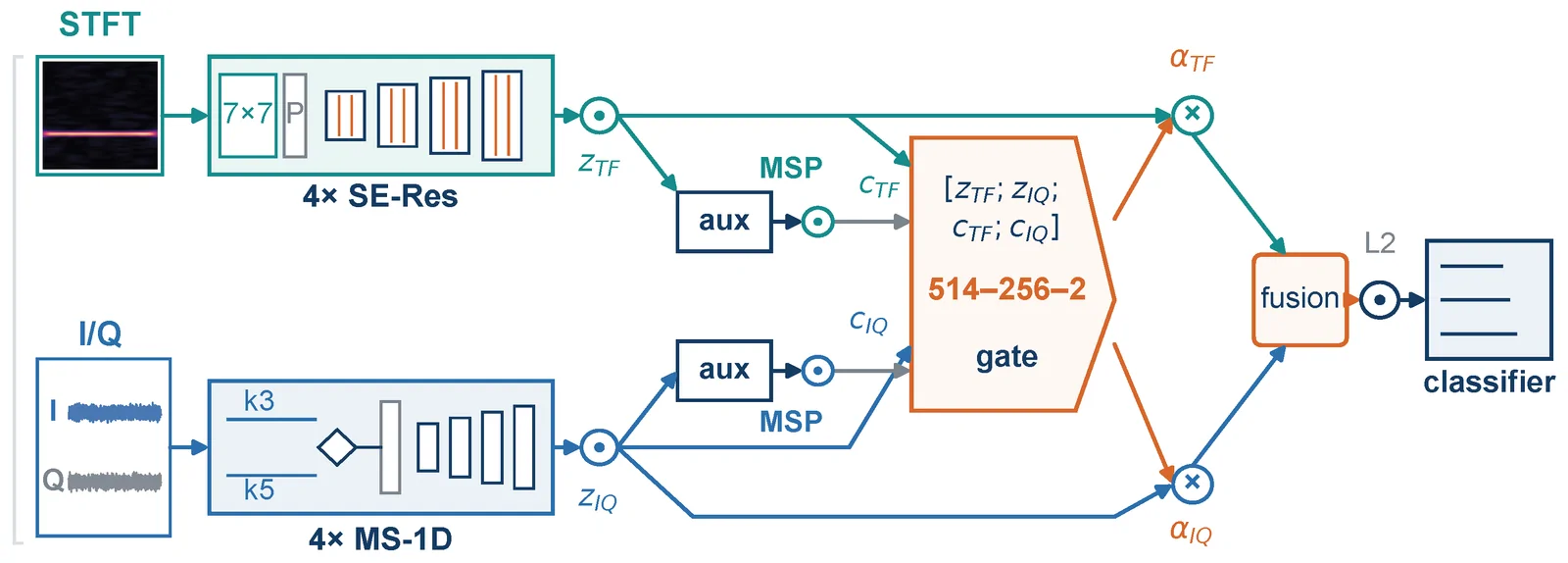
Dual-view encoder and sample-wise gate used by MM-CILNet. The STFT branch produces $z^{\mathrm{TF}}$ through a 7×7 stem, pooling, and four SE-residual stages, while the I/Q branch produces $z^{\mathrm{IQ}}$ through four multi-scale 1-D stages with k=3 and k=5 branches. The gate forms $\left[z^{\mathrm{TF}};z^{\mathrm{IQ}};c_{\mathrm{TF}};c_{\mathrm{IQ}}\right]$, where $c_{\mathrm{TF}}$ and $c_{\mathrm{IQ}}$ are maximum-softmax cues from the representation-specific auxiliary heads, and a 514–256–2 MLP yields $\left(\alpha^{\mathrm{TF}},\alpha^{\mathrm{IQ}}\right)$. The weighted sum is $\ell_{2}$-normalized before the fused classifier. During the head-only post stage, both encoders and the gate are frozen while the fused, STFT-auxiliary, and I/Q-auxiliary classifier heads are updated.

Figure 2 shows the dual-encoder architecture of MM-CILNet. The I/Q encoder receives the two-channel I/Q sequence and applies four sequential multi-scale one-dimensional blocks with output widths 32, 64, 128, and 256. Within each block, parallel convolution–batch-normalization–ReLU branches with kernel sizes k∈{3,5} are concatenated, normalized and activated, and then downsampled by max pooling. Global average pooling and a linear projection produce a 256-dimensional output, which is $\ell_{2}$-normalized to obtain $z^{\mathrm{IQ}}\in R^{256}$.

The time-frequency encoder processes the STFT magnitude image with a lightweight squeeze-and-excitation residual backbone. A 7×7 stride-2 convolutional stem with batch normalization and ReLU is followed by 3×3 stride-2 max pooling and four residual stages with channel widths 32, 64, 128, and 256. Each stage contains two residual blocks, each comprising two 3×3 convolutions and a squeeze-and-excitation unit; the first block in stages 2–4 performs stride-2 downsampling. Global average pooling and a linear projection produce a 256-dimensional output, which is $\ell_{2}$-normalized to obtain $z^{\mathrm{TF}}\in R^{256}$.

### 3.3. Sample-Wise Gated Fusion

The embeddings $z^{\mathrm{IQ}}$ and $z^{\mathrm{TF}}$ are fused by a sample-wise gate. Each representation has a cosine auxiliary classifier. Its scalar score cue is the maximum softmax probability, $c_{v}=\max_{k}\mathrm{softmax}{\left(\ell_{v}\right)}_{k}$, for v∈{IQ,TF}. Following the implementation order, the gate forms $g=\left[z^{\mathrm{TF}};z^{\mathrm{IQ}};c_{\mathrm{TF}};c_{\mathrm{IQ}}\right]\in R^{514}$. A two-layer MLP (514–256–2, ReLU between layers) produces two logits, which are softmax-normalized as $\left(\alpha^{\mathrm{TF}},\alpha^{\mathrm{IQ}}\right)$; output indices 0 and 1 therefore weight the time-frequency and I/Q embeddings, respectively. The fused embedding is $z=\mathrm{norm}(\alpha^{\mathrm{TF}}z^{\mathrm{TF}}+\alpha^{\mathrm{IQ}}z^{\mathrm{IQ}})$ and is passed to the fused cosine classifier.

The concatenated embeddings carry representation-specific signal content and feature geometry, including agreement and conflict between the two views. The two maximum-softmax values summarize how sharply each representation-specific head separates its current class scores and enter the gate only as scalar context. During normal incremental training, both encoders, both auxiliary heads, the fused head, and the gate are optimized jointly with

(1) $$L_{\mathrm{train}}={\mathrm{CE}}_{\mathrm{fused}}+0.2\left({\mathrm{CE}}_{\mathrm{IQ}}+{\mathrm{CE}}_{\mathrm{TF}}\right),$$

allowing the MLP to learn interactions between feature content and score sharpness. The maximum-softmax values condition the representation mixture; they are not the predictive-entropy variable used in the post hoc stratified analysis.

The softmax outputs $\left(\alpha^{\mathrm{TF}},\alpha^{\mathrm{IQ}}\right)$ are learned sample-wise mixing coefficients. Direct Gate-versus-Mean comparisons in Section 5.3 evaluate whether this adaptive mixture improves classification over uniform averaging under AWGN, fading, and fading-plus-impulsive noise.

Fusion is applied after global pooling so that every fusion control combines the same two fixed-dimensional representations. The I/Q branch has a one-dimensional temporal axis, whereas the STFT branch has two-dimensional time–frequency bins; these positions are branch-specific and do not define a common spatial or temporal index over which one shared attention rule can be applied. An encoder-internal attention module would therefore change feature extraction, capacity, and the intervention location instead of isolating the fusion rule. The matched comparator applies one shared 256–128–1 Tanh scorer to the two pooled representation tokens and softmax-normalizes the two scores before weighted summation. Section 5.3 compares this representation-level attention, Gate, and Mean at the common fusion interface.

### 3.4. Post-Session Classifier-Head Fine-Tuning

After each incremental session’s joint training on current-session and replay data, the default MM-CILNet post stage freezes both encoders and the gate and updates three cosine classifier heads: the fused head and the I/Q- and STFT-specific auxiliary heads. It uses one epoch of cross-entropy training at $2\times 10^{-4}$ (0.2 times the main learning rate) on the updated 400-example class-balanced memory. With batch size 16, this is 25 optimization steps and 400 processed examples per incremental session. This operation changes decision boundaries but does not change the inference architecture. Section 5.3 also evaluates head-only sampling from the full current-session-plus-replay pool, full-model class-balanced fine-tuning, and full-model sampling from that same pooled training set with the same example and step budget.

### 3.5. Training Procedure

All models are trained using the Adam optimizer with an initial learning rate of $1\times 10^{-3}$ and weight decay of $1\times 10^{-4}$. The batch size is 16. The base session ($S_{0}$) trains for 5 epochs; each incremental session ($S_{1}$–$S_{6}$) trains for 3 epochs. These hyperparameters are held constant across MM-CILNet and all compared baselines. The main replay memory budget is 400 samples, updated to remain class-balanced over seen classes after each session.

Table 2 separates inference overhead from post-stage training overhead. The CPU benchmark used an AMD Ryzen 7 7735HS processor (Advanced Micro Devices, Inc., Santa Clara, CA, USA), one PyTorch 2.6.0 CPU thread, batch size 1, 10 warm-up passes, and 50 timed forward passes; latency is the arithmetic mean per sample. Gated fusion takes 22.2 ms, 3.5% longer than Mean fusion (21.4 ms). The current shared model container serializes 3.502 M parameters because it instantiates all fusion controls, whereas the executed forward paths contain 3.272 M parameters for Gate and 3.140 M for Mean. At K=20, the post-session stage updates only the three classifier heads (15,360 parameters). Removing dormant controls gives 3.272 M parameters for a Gate deployment; a Mean-only fused-prediction deployment can additionally remove the two unused auxiliary heads, giving 3.130 M.

Let $C_{\mathrm{IQ}}$ and $C_{\mathrm{TF}}$ denote the forward costs of the two encoders, *d* the embedding dimension, *h* the gate hidden width, *K* the number of seen classes, and *M* the post-stage sample count. MM-CILNet inference has complexity $O(C_{\mathrm{IQ}}+C_{\mathrm{TF}}+\left(2d+2\right)h+2h+3dK)$: the first two terms are shared by all dual-representation comparisons, the middle terms are the two-layer gate, and the final term covers the fused and two auxiliary cosine heads. Mean fusion removes the gate term. The gate MLP contains 132,354 parameters, the two-token attention scorer contains 33,025, and the two auxiliary heads contain 10,240 at K=20. For d=h=256, the Gate overhead is small relative to the encoders, which is consistent with the measured 3.5% CPU latency increase. One head-only post stage costs $O(M\left[C_{\mathrm{IQ}}+C_{\mathrm{TF}}+\left(2d+2\right)h+3dK\right])$ in forward computation and O(MdK) in trainable-head backpropagation; it does not backpropagate through the encoders or gate. Full-model post-stage controls have the same forward order but additionally backpropagate through both encoders and the gate. Thus, relative to the common replay-style adaptations, the leading encoder complexity is unchanged, and the additional costs are confined to the lightweight gate and one bounded post-session pass.

For a direct comparison with the implemented CIL families, define $B=C_{\mathrm{IQ}}+C_{\mathrm{TF}}$, G=(2d+2)h+2h, and H=3dK. Table 3 lists method-specific additions beyond the common current-plus-replay update. All shared-backbone Gate adaptations have the same encoder-dominated inference order O(B+G+H); their differences occur during incremental training or post-session processing. Compared with the distillation families, MM-CILNet avoids a frozen previous-network forward pass; compared with DER-style replay, it avoids storing and matching old logits. Its additional work relative to plain replay is the bounded M=400 head-only pass.

## 4. Experimental Setup

### 4.1. CIL-20 Dataset and Protocol

We construct CIL-20, a 20-class radar intra-pulse modulation dataset under an 8+2×6 class-incremental protocol. The base session $S_{0}$ contains 8 classes; each subsequent session $S_{1}$–$S_{6}$ introduces 2 new classes, with all 20 classes appearing by $S_{6}$. After each session, evaluation covers all classes observed so far, matching the CIL condition that task identity is unavailable at test time.

CIL-20 includes both basic modulation families and compound modulation types, increasing the difficulty of unified recognition after continual class expansion. The 20 classes span four modulation families: Basic (NS, LFM, Barker), Phase (Frank, P1, P2, P3, P4), Time/Frequency (Costas, T1, T2, T3, T4, NLFM, HFM, SFM), and Compound (BPSK-LFM, Frank-LFM, P4-LFM, SF-PC). Note that Costas codes are frequency-hop waveforms and are classified within the Time/Frequency family, which is consistent with their time-frequency coding structure. Table 4 summarizes the dataset and protocol parameters used in the main experiments.

Each clean signal is normalized to unit root-mean-square amplitude, corrupted by complex AWGN at the selected SNR, and normalized again. A single NumPy generator seeded at 42 advances for every generated example. Train, validation, and test files are generated in separate loop iterations and have disjoint file identifiers and noise draws. Classes with randomized waveform parameters also receive independent parameter draws across splits; classes defined by a fixed phase-code formula differ across splits primarily through independently generated noise. Thus, the split does not reuse one clean waveform with three noise labels for randomized classes, but it is not a source-separated measured-signal split.

Table 5 summarizes the clean-waveform parameters. Frequencies and bandwidths are fractions of the 100 MHz sampling rate $f_{s}$; $T_{p}=1024/f_{s}$ is the pulse duration. Compound classes multiply the corresponding independently generated component waveforms before normalization.

For the STFT view, the complex signal is Fourier-resampled from 1024 to 256 samples. SciPy’s STFT uses its default Hann window, $n_{\mathrm{perseg}}=64$, $n_{\mathrm{overlap}}=48$, $n_{\mathrm{FFT}}=64$, two-sided output, zero boundary extension, and padding. The magnitude is min–max scaled per sample, bilinearly resized to 224×224, and clipped to [0,1]. Continual class expansion then follows the 8+2×6 protocol. A fixed 400-example replay memory is updated by seeded random selection to remain class-balanced over seen classes. All compared methods use the same class order, memory budget, data split, and preprocessing unless stated otherwise.

Beyond the main CIL-20 protocol, we run distribution-shift and external validation experiments. The shift tests include fading and stronger fading-plus-impulsive-noise conditions. Three radar-related datasets provide related-task transfer checks with different signal sources, label spaces, and recognition targets. Table 6 defines the evidential role of each dataset and distinguishes these transfer checks by signal source, label space, and recognition task.

### 4.2. Compared Methods

MM-CILNet is compared with a direct replay baseline and representative CIL-style adaptations. Replay uses the same dual-representation backbone and class-balanced memory update as MM-CILNet but omits the post-session stage. This comparison estimates the complete-protocol effect; the matched controls in Section 5.3 separately identify additional optimization, parameter scope, and sampling.

The “-style” label denotes in-house adaptations under the common MM-CILNet training pipeline. The dual-representation backbone, random class-balanced memory update, data split, class order, and seed set remain fixed. iCaRL-style uses replay plus output distillation (weight 1, temperature 2); BiC-style uses the same distillation plus one class-balanced post-session head update in place of the official held-out validation layer; PODNet-style adds prototype regularization (weight 0.1) to distillation; DER-style matches stored replay logits with MSE (weight 1); DER+head-FT-style adds the same post-session head update to DER; and WA-style performs post-hoc old/new classifier-weight norm alignment. The resulting table is a controlled mechanism-family comparison under one signal-processing and training path; performance of the official methods remains defined by their original implementations and protocols.

We focus on backbone-agnostic replay, bias-correction, distillation, and weight-alignment families because they can share the signal input and memory protocol. The common base schedule is 5 epochs for $S_{0}$ and 3 epochs per incremental session. Method-specific loss weights and post-hoc operations are retained as listed above. The reported results characterize these adaptations under the shared configuration; method-specific optimization is reserved for direct reproduction of each official implementation.

The ablation study separates representation, fusion, post-stage parameter scope, sampling, and additional optimization. All matched-control conditions use the same seeds, 400 post-stage examples, and 25 post-stage mini-batches whenever the post stage is enabled. Table 7 summarizes the shared conditions and adaptation-specific differences used to ensure a controlled comparison.

### 4.3. Evaluation Metrics and Statistical Tests

The primary metrics are All accuracy, Old accuracy, New accuracy, average forgetting, macro-F1, and the old/new bias gap. All accuracy is computed over all classes observed up to the current session. Old accuracy is computed over classes introduced in previous sessions, and New accuracy over classes introduced in the current session. Together, these metrics distinguish unified recognition, old-class retention, and new-class learning.

Let $a_{j,t}$ denote the test accuracy on the classes introduced in session *j* after training through session *t*. At final session *T*, forgetting for session j<T is

(2) $$F_{j,T}=\max_{t\in \{j,\ldots ,T\}}a_{j,t}-a_{j,T},$$

and average forgetting is ${\bar{F}}_{T}=T^{-1}\sum_{j=0}^{T-1}F_{j,T}$. The statistical unit is one random-seed run; the manuscript reports the mean and standard deviation of ${\bar{F}}_{T}$ over seeds. Macro-F1 is computed over all seen classes at the final session.

The final-session absolute old/new bias gap is

(3) $$G_{T}=\left|A_{T}^{\mathrm{old}}-A_{T}^{\mathrm{new}}\right|,$$

where both accuracies are sample-level accuracies for one seed at $T=S_{6}$. We compute $G_{T}$ per seed before paired testing or seed aggregation. A smaller gap alone is not considered an improvement; it is interpreted jointly with All, Old, New, and forgetting.

All main statistical comparisons use random seed as the statistical unit. For CIL-20, results are reported over 12 matched seeds as mean ± sample standard deviation. Paired contrasts report the two-sided paired *t*-test, a Wilcoxon signed-rank sensitivity analysis, Cohen’s $d_{z}$, and a 95% paired-difference interval. Holm correction is applied within each explicitly declared family of multiple matched contrasts. Table 8 contains one prespecified Replay–MM-CILNet contrast per endpoint, so its paired *p*-values are unadjusted across endpoints; exploratory unadjusted results are labeled as such. The complete 4×2 fusion-by-head-fine-tuning and 2×2 trainable-scope-by-sampling designs are analyzed with repeated-measures ANOVA. The threshold is p<0.05.

### 4.4. Implementation Details

MM-CILNet uses two representation-specific encoders. A multi-scale one-dimensional convolutional branch extracts I/Q waveform features, and a lightweight two-dimensional convolutional branch extracts STFT features. Both outputs are mapped to 256 dimensions and fused by the sample-wise gate.

The gate is the 514–256–2 multilayer perceptron defined in Section 3.3. The fused feature is fed into a cosine classifier over all seen classes, and the encoders, gate, and three classifier heads are optimized jointly during the main incremental update.

All models are trained using the Adam optimizer with an initial learning rate of $1\times 10^{-3}$ and a weight decay of $1\times 10^{-4}$. The batch size is set to 16. The base session is trained for 5 epochs, and each incremental session is trained for 3 epochs. These settings are kept the same for the main method and compared baselines unless otherwise specified.

After each incremental session, the default post stage freezes both encoders and the gate and updates the fused, I/Q, and STFT classifier heads using the updated class-balanced memory. Fine-tuning uses one epoch and a learning rate scaled by 0.2, giving $2\times 10^{-4}$.

All compared methods are trained under the same data split, memory budget, class order, and random seed setting. The main memory budget is fixed to 400 samples unless otherwise specified. The same preprocessing pipeline is used to generate I/Q and time-frequency inputs for all methods.

Formal GPU training used Python 3.12.0, PyTorch 2.6.0 with CUDA 12.4, NumPy 2.4.4, and SciPy 1.17.1. The final statistical analyses, CPU benchmarks, and manuscript figures used Python 3.11.4, PyTorch 2.6.0, NumPy 1.26.4, SciPy 1.15.1, pandas 2.2.3, statsmodels 0.14.4, and Matplotlib 3.10.0. The accompanying reproducibility materials contain the generator, fixed protocol and metadata files, matched-control configurations, resolved experimental settings, sampling records, seed-level metrics, and analysis scripts.

## 5. Results and Analysis

The experiments first establish the gain of the complete MM-CILNet protocol and then identify the design variables that control it. Equal-budget controls show significant post-session effects on All and Old accuracy across fusion rules, while trainable scope and sampling select distinct retention–plasticity operating points. This positions MM-CILNet as a protocol-level contribution whose representation, replay, and post-session components must be designed jointly.

### 5.1. Main CIL-20 Results

We first evaluate MM-CILNet on the main CIL-20 protocol. Unlike closed-set radar modulation recognition, the class-incremental setting requires unified prediction over all classes observed so far after each session. The analysis therefore considers final All accuracy alongside old-class retention, new-class recognition, and old/new balance.

**Figure 3:**
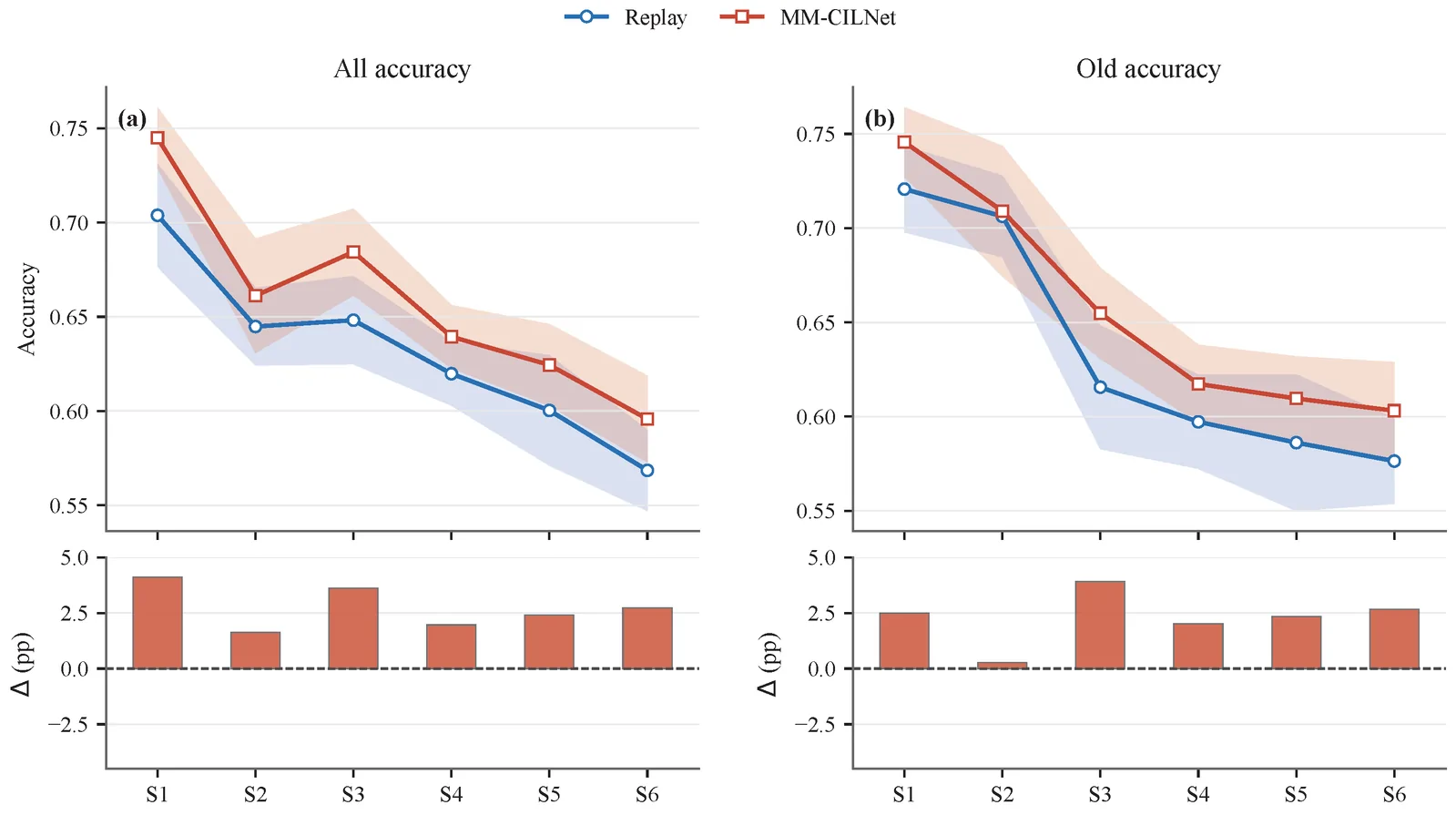
Session-wise All and Old accuracy for Replay and MM-CILNet on CIL-20 ($S_{1}$–$S_{6}$). Panel (**a**) shows All accuracy, and panel (**b**) shows Old accuracy. Lines show 12-seed means and shaded bands show ±1 sample standard deviation. Lower panels show the matched-seed mean Δ=MM-CILNet−Replay in percentage points.

Figure 3 shows that MM-CILNet maintains higher All and Old accuracy across incremental sessions. The delta bars confirm a consistent positive gap that emerges after class expansion begins and persists through the final session, indicating improved old-class retention.

Table 8 reports the paired comparison at $S_{6}$ under the 8+2×6 protocol. MM-CILNet improves All accuracy by +2.73 percentage points (95% CI [1.11,4.34]; p=0.0034) and Old accuracy by +2.67 percentage points (95% CI [0.77,4.58]; p=0.0103). Macro-F1 improves by +2.73 percentage points (95% CI [1.04,4.41]; p=0.0045). Because the final test covers all 20 classes, uses a fixed 400-sample memory, and includes SNRs down to −18 dB, these gains reflect accumulated behavior during class expansion rather than a high-SNR closed-set effect.

The paired tests confirm this interpretation. With matched random seeds, the paired *t*-test yields p=0.0034 for All accuracy and p=0.0103 for Old accuracy. The Wilcoxon signed-rank sensitivity analysis yields p=0.0054 and p=0.0122, respectively. The paired effect sizes are substantial, with Cohen’s $d_{z}=1.0743$ for All accuracy and $d_{z}=0.8919$ for Old accuracy. Differences in All and Old accuracy are positive in 10 of the 12 matched seeds, and the Macro-F1 difference is positive in 11 of 12. The magnitude, paired intervals, and seed directions therefore support a repeatable efficacy gain without implying that every seed improves.

The strongest and most stable effects are concentrated in All accuracy, old-class retention, and macro-F1. Average forgetting is reported descriptively: MM-CILNet has the lowest mean among the four methods with recoverable histories in the baseline comparison, but its Replay contrast changes sign between the two independent matched-seed cohorts analyzed below.

Final-session accuracy should be interpreted under the deliberately difficult protocol, which evaluates all 20 classes seen across sessions, 16 SNR levels down to −18 dB, limited per-class samples, and a fixed replay memory.

### 5.2. Comparison with Representative Class-Incremental Baselines

We next compare MM-CILNet with iCaRL-style, BiC-style, PODNet-style, DER-style, DER+head-FT-style, and WA-style adaptations. All methods use the same dual-representation input setting, session split, memory budget, base schedule, and random seed set, with adaptation-specific losses and post-hoc operations described in Section 4.2.

Table 9 reports the final-session CIL-20 results. Under the shared configuration, MM-CILNet achieves the best All and Old accuracy among the listed adaptations and the lowest average forgetting among the four methods for which the session-history records are available. Table 10 provides the mechanism attribution under matched post-session budgets.

BiC-style obtains the highest New accuracy but is worse in All accuracy, Old accuracy, and average forgetting. For radar CIL, a strong response to new classes can still produce a poorer unified classifier. The target is the balance among new-class learning, old-class retention, and old/new bias.

Table 8 and Table 9 report separate 12-seed experiments for paired efficacy and multi-baseline comparison, respectively. Their small numerical differences reflect independent training realizations, while both locate MM-CILNet’s advantage in all-class recognition and old-class retention.

### 5.3. Ablation Analysis

The matched-control experiment uses 11 conditions and 12 matched seeds. In every enabled post stage, 400 examples are processed in 25 mini-batches. “Balanced” samples an equal number per seen class from the updated memory; “Current+replay” draws a uniform 400-example subset without replacement from the full current-session-plus-replay training pool. “Heads” updates the fused, I/Q, and STFT classifier heads, whereas “Full” updates the complete network. Across conditions, the data split, normal-training sample streams, optimizer, schedule, checkpoint rule, and evaluation set are fixed. Only representation, post-stage trainable scope, and post-stage sampling vary as specified above.

Table 11 reports the paired head-only-minus-none differences for all four representation rules. The corresponding condition means and sample standard deviations appear in Table 10; the intervals below are computed directly from the 12 within-seed differences.

The primary Replay comparison in Table 8 and the Gate controls in Table 10 are independently trained 12-repeat experiments. Conditions are paired only within each experiment, and all intervals and tests are therefore computed within each experiment. Both experiments show positive All/Old differences (+2.73/+2.67 pp and +1.50/+1.26 pp, respectively); the forgetting difference changes from −0.96 pp to +1.65 pp and is not pooled across experiments.

The 4×2 repeated-measures analysis (Gate, Mean, I/Q, and STFT; with and without balanced head fine-tuning) finds main effects of fusion ($p=5.74\times 10^{-15}$ for All; $2.00\times 10^{-16}$ for Old) and head fine-tuning ($p=2.14\times 10^{-4}$ for All; $4.66\times 10^{-4}$ for Old), without interactions (p=0.9518 and 0.5407). The New-accuracy main effects and interaction are not significant. Forgetting shows a fusion-by-fine-tuning interaction ($p=1.30\times 10^{-4}$), so the direction of the forgetting change depends on representation.

Within Gate fusion, G–HB improves All by 1.50 pp relative to G–N, but its paired interval includes zero (95% CI [−0.26,3.26] pp; unadjusted p=0.0872; Holm-adjusted p=0.3488). Its Old difference is +1.26 pp (95% CI [−0.72,3.25] pp; p=0.1882). The Gate-only 2×2 design finds trainable-scope effects on All and Old (p=0.0239 and 0.0152), a sampling effect and interaction on New (p=0.00405 and 0.00341), and scope, sampling, and interaction effects on forgetting. G–FB yields the lowest forgetting but also the lowest New accuracy; G–FN yields the highest All and New accuracy. Head-only fine-tuning is therefore a bounded-cost operating point within a broader scope–sampling trade-off, rather than the sole optimum for every endpoint.

Gate and Mean with balanced head fine-tuning have nearly identical All accuracy (difference +0.02 pp; 95% CI [−1.51,1.55] pp; p=0.9805). The same pattern holds in the checkpoint cross-evaluation under channel shift reported in Table 12:

A post hoc stratified analysis finds systematic coefficient differences across predictive-entropy and class-similarity groups. Predictive entropy defines the analysis strata and is separate from the gate inputs. Together with the direct Gate–Mean comparison above, this result describes variation in the learned representation mixture without assigning the coefficients a separate performance meaning.

At the same pooled-embedding interface, a lightweight representation-level attention control obtains final-session All, Old, and New accuracies of 0.5806±0.0198, 0.5922±0.0222, and 0.4766±0.0738, respectively, over the same 12-seed protocol. This control evaluates learned attention over the two representation tokens. A single spatial or temporal attention map is not a like-for-like fusion rule because the I/Q branch indexes one-dimensional time samples whereas the STFT branch indexes two-dimensional time–frequency bins. Placing attention inside either encoder would also change feature extraction and parameter capacity. The two-token control therefore answers the fusion question at the common interface while holding the encoder architectures, output dimensions, and pooled-embedding interface fixed.

### 5.4. Per-Family Descriptive Analysis

To examine where the complete-protocol difference occurs across modulation families, we pool the final-session confusion matrices over the same 12 seeds for Replay and MM-CILNet. Each matrix contains 48 test examples per class and seed (11,520 predictions per method). The resulting family accuracies localize the pooled prediction differences; seed-level family statistics would be required for family-specific inference.

Table 13 reports the pooled family-level comparison. The pooled comparison is positive in all four predefined families, with the largest descriptive differences in Basic (+0.0544) and Compound (+0.0482). The Phase difference is small (+0.0080). These values localize the aggregate outcome within CIL-20, while mechanism attribution comes from the seed-level matched controls in Section 5.3.

### 5.5. Old/New Class BIAS Analysis

In class-incremental learning, degradation appears not only as lower old-class accuracy but also as old/new bias. During an incremental session, new classes dominate optimization while old classes are represented only by replay samples, allowing the classifier to shift toward new-class regions.

We quantify this behavior with the absolute old/new bias gap. A smaller gap means a more balanced classifier across old and new classes. As shown in Table 14, Replay has a gap of 0.1887 on the main CIL-20 protocol, while MM-CILNet reduces it to 0.1535. This is an absolute reduction of 0.0352, or approximately 18.7%, with a paired-test value of p=0.0297 and an effect-size magnitude of |d|=0.7206.

This paired outcome establishes a more balanced final classifier under the complete protocol. Component attribution is supplied by the matched scope–sampling controls in Table 10.

Confusion-level analysis is consistent with this mechanism. Replay tends to push old-class samples into later-introduced classes when phase-coding structures, frequency trajectories, or compound patterns are similar. MM-CILNet reduces cross-session confusion in several family-level error patterns.

**Figure 4:**
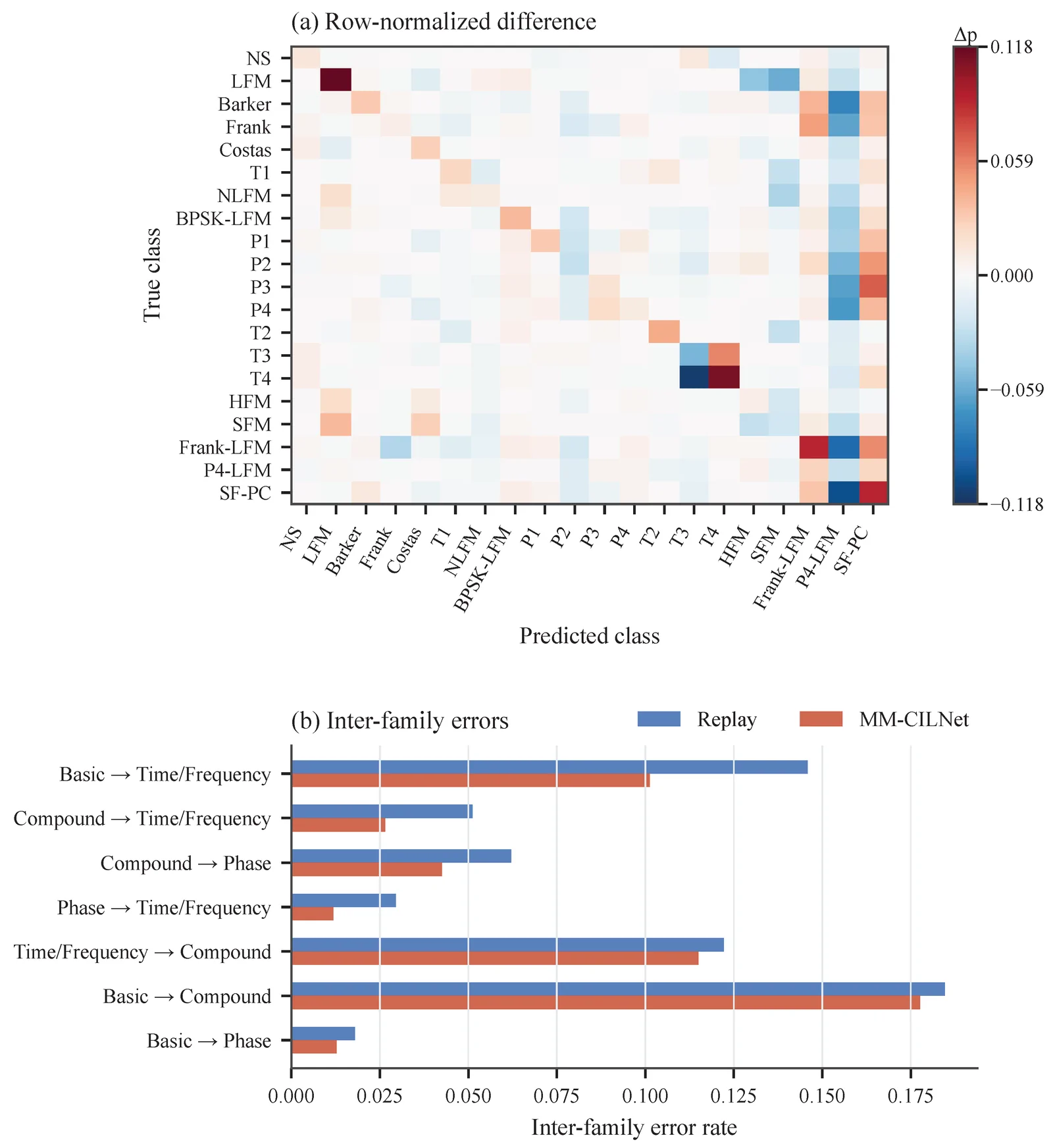
Final-session confusion changes on CIL-20. (**a**) Row-normalized MM-CILNet-minus-Replay difference pooled over 12 matched seeds (48 test examples per class and seed); blue off-diagonal cells indicate fewer errors. (**b**) Inter-family error rates for source–prediction pairs reduced by MM-CILNet. The family partition follows Table 13; seed-level inference uses whole-run metrics.

Figure 4 uses the same 12-seed pooled confusion matrices as Table 13. For off-diagonal cells, blue indicates lower error under MM-CILNet and red indicates higher error; diagonal cells have the opposite performance interpretation because they represent correct classifications. The largest visible off-diagonal structures occur among phase-coded classes and among frequency-coded classes. Panel (b) lists the source-to-predicted family pairs with positive descriptive Replay-to-MM-CILNet error-rate reductions; it is not an inferential ranking. The pooled confusion matrices and generation code are included in the accompanying reproducibility materials.

### 5.6. SNR-Stratified Analysis

We examine how the complete-protocol MM-CILNet–Replay difference varies across SNR levels at the final session. Table 15 reports matched 12-seed means and sample standard deviations for both methods at every evaluated SNR.

**Figure 5:**
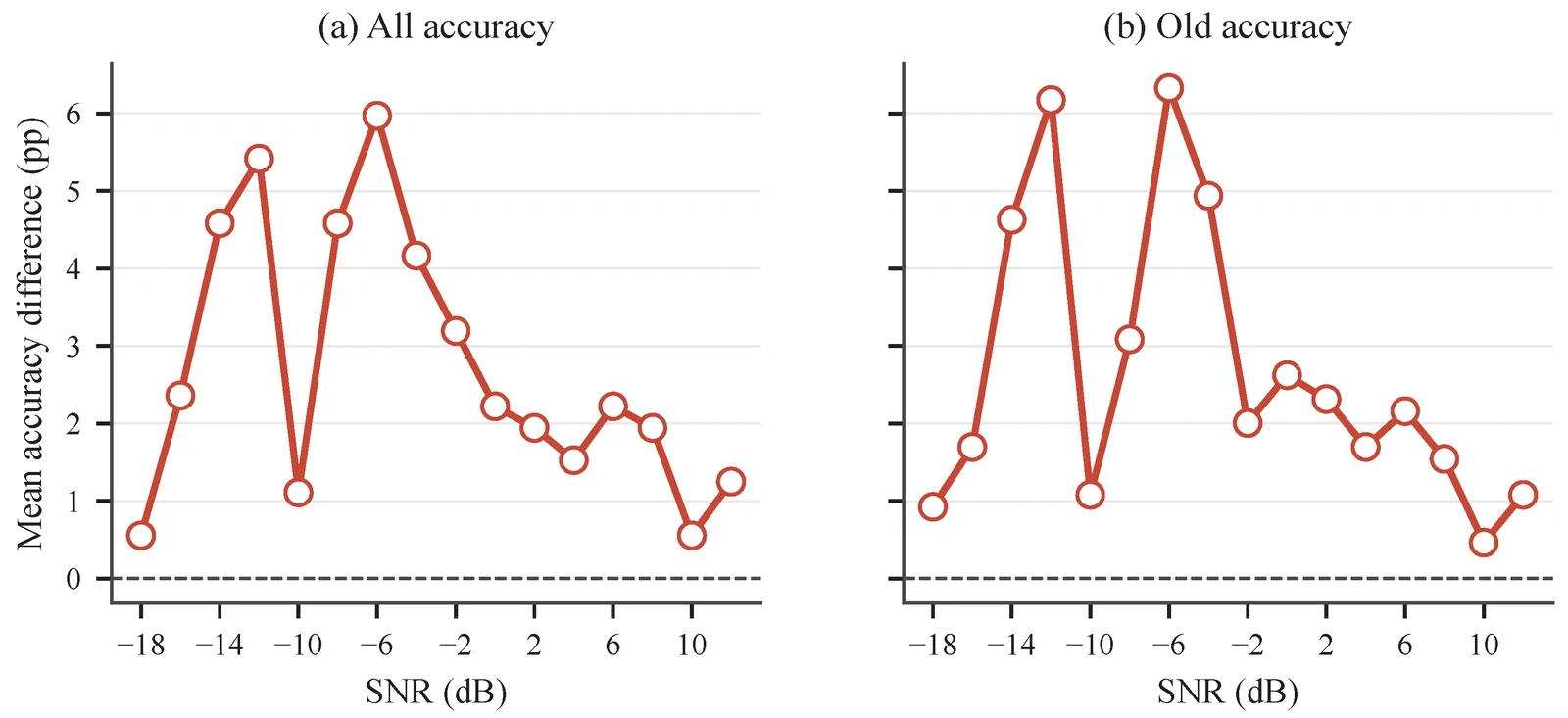
SNR-stratified final-session accuracy difference on CIL-20. Points show Δ=MM−CILNet−Replay mean differences over 12 matched seeds at the 16 measured SNR levels. Connecting lines indicate the ordered SNR sweep only and do not interpolate unmeasured SNRs or imply per-SNR inference. The horizontal dashed line denotes equal mean accuracy.

Figure 5 shows the SNR-stratified delta accuracy at $S_{6}$. The largest descriptive mean differences occur from −12 to −2 dB, whereas both conditions approach the noise floor at −18 to −14 dB. The points summarize the tested SNR levels and are not separate per-SNR inferential comparisons. This figure is a complete-protocol sensitivity analysis; component attribution is provided by the equal-budget controls in Section 5.3.

### 5.7. Distribution Shift and External Validation

We further evaluate the complete MM-CILNet protocol under distribution shift and on related external datasets. These experiments measure outcome consistency across altered channels, signal sources, label spaces, and recognition tasks.

Under the fading-shift condition, MM-CILNet yields a statistically supported improvement: final-session All accuracy rises from 0.2917 to 0.2980 (p=0.0421), and Old accuracy rises from 0.2878 to 0.2959 (p=0.0352). The complete post-session protocol remains beneficial when the shifted environment leaves the representation partially discriminative.

**Figure 6:**
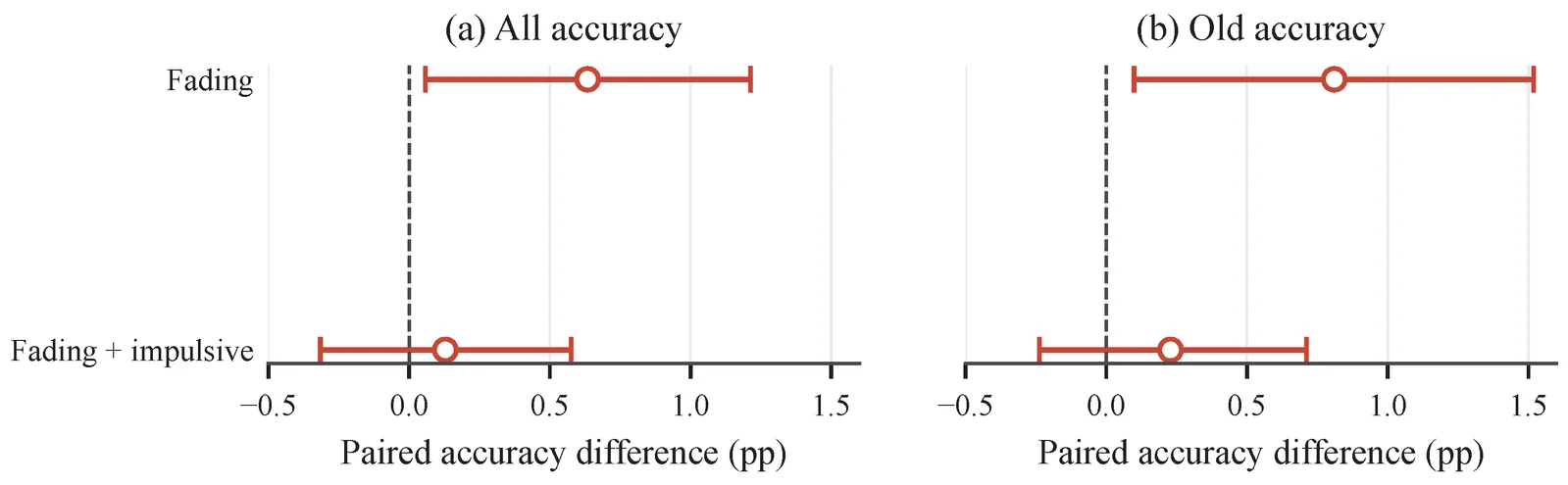
Distribution-shift paired effects at $S_{6}$. Points show mean paired differences, Δ=MM−CILNet−Replay, over 34 matched repeats; horizontal bars show paired bootstrap 95% confidence intervals. The vertical dashed line marks zero difference. Intervals crossing zero leave the effect direction unresolved at the tested repeat count.

Under stronger fading-plus-impulsive noise, the MM-CILNet–Replay difference is positive but small, and the direct Gate-versus-Mean comparison in Table 12 is statistically indistinguishable. The statistically supported shifted-channel gain is therefore localized to the fading condition reported above. Figure 6 summarizes the paired effects under the two shift conditions.

For an external measured check, we use a public communication-I/Q dataset from Mendeley Data [34]. Seven modulation classes are organized into a 3+2+2 protocol. Table 16 reports the final-session results. MM-CILNet achieves the best final All accuracy, Old accuracy, and average forgetting among the compared methods, matching the main CIL-20 pattern of improved old-class retention and overall balance.

The NTIA compound radar waveforms and Radar-PRI data provide transfer checks with different label spaces and sensing protocols [35,36]. Table 17 and Table 18 report outcomes under the adapted method set and establish executable performance on these related tasks. Mechanism transfer is evaluated separately below. For Radar-PRI, the adapted-method outcome suite contains 24 seeds (42–65), whereas the paired mechanism test uses the 17 seeds (42–58) for which both Replay-only and Replay-plus-head-fine-tuning records are available; seeds without both arms are excluded from the paired estimand rather than imputed or analyzed as unpaired observations.

### 5.8. External Outcome and Mechanism Checks

Table 19 summarizes the descriptive Old-accuracy difference between MM-CILNet and the strongest listed adaptation in each setting. These task-specific outcome comparisons complement the matched mechanism tests reported in Table 20.

To test the post-stage mechanism directly, Table 20 compares matched Replay-only and Replay-plus-head-fine-tuning runs. Real7 shows a statistically supported effect; NTIA and Radar-PRI provide related-task outcome checks without a statistically resolved paired effect.

### 5.9. Sensitivity Analysis

We examine how the complete post-stage protocol difference changes with memory budget and class order, and then test whether an intentionally recent-class-heavy replay memory changes the isolated effect of head-only fine-tuning. Table 21 and Table 22 report the memory-budget and class-order sensitivity results, respectively.

At 200 samples, the paired Old-accuracy difference is +0.07 pp (95% CI [−1.55,1.68] pp; p=0.9284), so an advantage is not detected at this budget. The difference is +2.67 pp at the default 400 samples (95% CI [0.77,4.58] pp; p=0.0103) and +1.86 pp at 800 samples (95% CI [0.26,3.46] pp; p=0.0265). Thus, the tested higher-budget points retain the Old-accuracy advantage, while the 200-sample point does not; these three discrete budgets establish budget dependence but do not locate a sharp threshold. Across five randomized class orders, the difference is positive in every tested order (mean +2.27 pp). These sensitivity checks characterize the complete training protocol, whereas the equal-budget matrix in Section 5.3 supplies component attribution.

To stress replay-memory balance, we crossed memory allocation (class-balanced versus recent-class-heavy) with the post stage (none versus head-only) under Gate fusion. The recent-class-heavy allocation assigned each current-session class a fixed weight 2.5 times that of each old class. Integer quotas were obtained by deterministic largest remainder, with current classes taking ties first and class ID resolving remaining ties; the resulting per-current-class quotas were 77, 67, 59, 53, 48, and 44 in $S_{1}$–$S_{6}$. In every session, 400 exemplars were sampled without replacement. The updated memory was used in normal incremental training and, when enabled, in the 25-step head-only post stage. The balanced cells reuse the 12-seed G–N and G–HB controls in Table 10; the two recent-class-heavy cells were newly evaluated with the same seeds 42–53 and otherwise matched scientific settings. Table 23 reports the resulting four matched conditions.

Under balanced memory, head-only fine-tuning changed Old accuracy by +1.26 pp (95% CI [−0.72,3.25] pp; p=0.188); under recent-class-heavy memory, it changed Old accuracy by +0.89 pp (95% CI [−0.39,2.16] pp; p=0.154). Relative to their balanced counterparts, recent-class-heavy replay changed Old accuracy by −1.63 pp without a post stage (95% CI [−3.23,−0.03] pp; unadjusted p=0.0466; four-endpoint Holm p=0.140) and −2.01 pp with the head-only stage (95% CI [−4.08,0.07] pp; unadjusted p=0.0571; Holm p=0.114). Average forgetting increased by +4.69 pp without a post stage (95% CI [2.62,6.75] pp; Holm p=0.0016) and +3.66 pp with the head-only stage (95% CI [1.01,6.32] pp; Holm p=0.0449). The prespecified seed-paired interaction (recent-heavy minus balanced) was −0.38 pp (95% CI [−3.24,2.49] pp; t(11)=−0.289, p=0.778, Cohen’s $d_{z}=-0.083$). A two-sided Wilcoxon signed-rank sensitivity analysis gave W=38 and p=0.970. The secondary interactions were −0.36 pp for All accuracy (95% CI [−2.55,1.84]), −0.17 pp for New accuracy (95% CI [−10.60,10.25]), and −1.02 pp for average forgetting (95% CI [−4.75,2.70]); all three Holm-adjusted *p*-values were 1.000. Taken together, the Old-accuracy and forgetting endpoints support class-balanced replay as the retention-oriented default at the tested 2.5:1 stress. The head-only stage raises the mean Old accuracy under both allocations, but neither simple effect is statistically resolved at 12 seeds and the update does not erase the observed retention deficit under recent-heavy replay.

## 6. Discussion

### 6.1. Engineering Value for Continual Radar Recognition

The central result is an operating gain under sequential class expansion rather than a closed-set accuracy increment. With only 400 replay examples for 20 seen classes and test SNRs extending to −18 dB, MM-CILNet improves both final All and Old accuracy by approximately 2.7 pp over matched Replay. Under the shared configuration, it also achieves the best All and Old accuracy among the listed radar-adapted CIL methods and the lowest average forgetting among the four methods with recoverable session histories. The practical value is therefore a radar recognition protocol that retains established waveform classes while continuing to absorb new ones from complementary same-pulse representations.

The same-pulse I/Q and STFT views play complementary roles in this protocol. The I/Q branch preserves phase and amplitude evolution, while the STFT branch exposes modulation trajectories in the time–frequency plane. Combining these representations with replay and a bounded decision-boundary update creates an intervention at the point where continual recognition is most vulnerable: the classifier must accommodate new classes without allowing recent data to erase older decision regions.

### 6.2. Attribution from the Matched Controls

The equal-budget decomposition turns the complete-protocol gain into concrete design choices. Across Gate, Mean, I/Q-only, and STFT-only representations, the post-session stage has significant main effects on All and Old accuracy. Within Gate fusion, trainable scope changes All and Old accuracy, while sampling changes the balance between New accuracy and forgetting. Full-model updates can match or exceed head-only All accuracy, but they occupy different retention–plasticity operating points. The resulting design principle is to select representation, trainable scope, and sampling jointly rather than treat classifier-head fine-tuning as an isolated universal mechanism.

The sample-wise gate provides a lightweight adaptive fusion interface. Direct Gate–Mean tests under AWGN, fading, and fading-plus-impulsive noise do not detect an All-accuracy difference. The post hoc coefficient strata show how the learned representation mixture varies across examples; the classification effect is determined by the direct Gate–Mean comparison.

### 6.3. Memory Allocation and Retention–Plasticity Control

The matched controls show why All, Old, New, and forgetting must be considered together. Full-model balanced fine-tuning maximizes Old accuracy and minimizes forgetting among the Gate post-stage conditions, whereas full-model current-plus-replay fine-tuning gives the highest All and New accuracy. The head-only balanced update occupies a bounded middle operating point and adds no inference parameters or operations.

The sensitivity experiments convert this trade-off into direct operating guidance. The complete protocol shows a positive, statistically detectable Old-accuracy difference at the tested 400- and 800-sample budgets and across all five tested class orders; at 200 samples, the +0.07 pp mean difference is not statistically detectable. These discrete budgets establish a budget-dependent effect without identifying a sharp threshold. Under the prespecified 2.5:1 recent-class-heavy allocation, Old accuracy falls by 1.63–2.01 pp and average forgetting rises by 3.66–4.69 pp relative to class-balanced replay. The direct operating conclusion is therefore to preserve class balance when old-class retention is the priority; the isolated one-epoch head update does not offset the retention cost of the tested allocation.

### 6.4. Transfer Scope

The distribution-shift and related-dataset experiments define the present transfer envelope of the complete protocol. Under fading, MM-CILNet improves All and Old accuracy relative to Replay. The matched head-fine-tuning effect is statistically supported on measured Real7 data, while NTIA and Radar-PRI provide executable outcome checks with different signal sources, label spaces, and recognition tasks. Together, these checks show that the implemented protocol is executable beyond CIL-20 and that the matched retention effect transfers to the measured Real7 setting, without treating the two radar-related proxy tasks as target-task replications.

CIL-20 enables matched-seed factorial analysis with controlled waveform parameters, but it uses synthetic signals and split files rather than independent measured emitters. The radar-adapted baselines use one shared schedule instead of independently tuned official implementations. These boundaries define the scope of the reported comparisons; they do not alter the matched CIL-20 result or the measured Real7 mechanism result.

## 7. Conclusions

MM-CILNet provides a practical multiview replay protocol for continual radar intra-pulse modulation recognition. By combining complementary same-pulse I/Q and STFT representations with class-balanced replay, sample-wise fusion, and a bounded post-session classifier update, it improves final-session All and Old accuracy over matched Replay by 2.73 and 2.67 percentage points, respectively. Under the shared configuration, it also achieves the best All and Old accuracy among the listed radar-adapted CIL baselines and the lowest average forgetting among the methods with recoverable session histories.

The controlled decomposition shows that representation, post-stage parameter scope, and sampling jointly determine the retention–plasticity operating point. The Old-accuracy advantage is retained at the tested 400- and 800-sample budgets and across all five tested class orders. Under 2.5:1 recent-class-heavy replay, Old accuracy decreases by 1.63–2.01 pp and forgetting increases by 3.66–4.69 pp relative to class-balanced replay. The resulting engineering guidance is direct: preserve class balance when old-class retention is the priority, and select the bounded post-session update as part of the complete multiview replay protocol rather than as a substitute for balanced memory.

## Figures and Tables

**Table 1:** Method-level distinction from the closest families. The rows identify the implemented intervention rather than claim equivalence to official code.

Method Family	Signal Input	Class-Expansion Mechanism	Distinguishing Intervention
Fixed-set radar multiview	Same-pulse I/Q and time–frequency views	None	Representation fusion under a fixed class set
iCaRL/PODNet-type CIL	Usually one learned representation	Exemplar replay with distillation or prototype constraints	Feature/output preservation across sessions
BiC/WA/LUCIR	Usually one learned representation	Replay or distillation with classifier rebalancing	Bias layer, weight alignment, or margin constraint
Cascaded gated classifier	Task-wise classifier outputs	Classifier cascade	Gates rescale classifier probabilities
MM-CILNet	Same-pulse I/Q and STFT views	Class-balanced replay with a bounded three-head update	Sample-wise embedding fusion plus matched representation/scope/sampling attribution

**Table 2:** Parameter scope and inference cost at K=20. Container includes every module instantiated by the shared implementation; executed is the current forward path; post-trainable counts the heads updated after a session; deployable is the minimal fused-prediction graph after dormant-module pruning. The CPU protocol is specified in the text.

Configuration	Container (M)	Executed (M)	Post-Trainable (M)	Deployable (M)	GFLOPs	Latency (ms)
Mean fusion + head FT	3.502	3.140	0.015	3.130	0.960	21.4
MM-CILNet (gated)	3.502	3.272	0.015	3.272	0.960	22.2

**Table 3:** Complexity of MM-CILNet versus the radar-adapted CIL families used in the controlled comparison. *N* is the number of normal current-plus-replay training examples in a session, and $K_{o}$ is the number of old classes. Entries list method-specific additions beyond the common trainable current-model update.

Adaptation	Inference	Additional Incremental-Stage Computation or Storage
Replay-style	O(B+G+H)	None beyond the common replay update
iCaRL-style	O(B+G+H)	Frozen previous-model forward, $O(N\left[B+G+dK_{o}\right])$
PODNet-style	O(B+G+H)	Previous-model forward plus prototype regularization, $O(N\left[B+G+dK_{o}+d\right])$
DER-style	O(B+G+H)	Logit matching $O(NK_{o})$ and replay-logit storage $O(MK_{o})$
WA-style	O(B+G+H)	Once-per-session classifier norm alignment, O(dK)
BiC-style adaptation	O(B+G+H)	Previous-model forward plus bounded head pass, $O(N\left[B+G+dK_{o}\right]+M\left[B+G+H\right]+MdK)$
MM-CILNet	O(B+G+H)	Bounded head pass, O(M[B+G+H]+MdK); no previous-model forward or stored logits

**Table 4:** CIL-20 dataset and protocol parameters used in the main experiments.

Item	Setting
Number of classes	20 radar intra-pulse modulation classes
Class-incremental protocol	8+2×6 sessions ($S_{0}$–$S_{6}$)
SNR range	−18 dB to 12 dB, step 2 dB
Samples per class/SNR	5 train, 2 validation, 3 test
Total split size	1600 train, 640 validation, 960 test samples
I/Q input	1024 complex samples at 100 MHz
Time-frequency input	256-point resampling; Hann-window STFT ($n_{\mathrm{perseg}}=64$, overlap 48, FFT 64); magnitude resized to 224×224
Replay memory	400 samples, class-balanced over seen classes

**Table 5:** CIL-20 clean-waveform generation settings. Uniform ranges denote independent draws for each generated example.

Class or Group	Generation Setting
NS	Carrier $f_{0}∼U\left(0.06,0.15\right)f_{s}$; initial phase $\phi_{0}∼U\left(0,2\pi \right)$.
LFM/NLFM/HFM	LFM bandwidth $U\left(0.03,0.12\right)f_{s}$ with random slope sign; NLFM bandwidth $U\left(0.03,0.12\right)f_{s}$ with the quadratic–sinusoidal law in the released generator; HFM bandwidth $U\left(0.03,0.10\right)f_{s}$.
SFM	Modulation frequency $U\left(2,10\right)/T_{p}$ and modulation index U(2,6).
Costas	One of three fixed eight-chip permutations; hop spacing $U\left(0.01,0.03\right)f_{s}$.
Barker/Frank	Barker length sampled from {2,3,4,5,7,11,13}; Frank matrix order sampled from {4,8}.
P1–P4/T1–T4	P1 and P2 use matrix order 8; P3 and P4 use code length 64; T1–T4 use 16 segments and the equations implemented in the released generator.
BPSK-LFM/Frank-LFM/P4-LFM/SF-PC	Products of the corresponding component waveforms; SF-PC uses eight frequency steps with spacing $U\left(0.01,0.025\right)f_{s}$ and a P4 phase code.

**Table 6:** External validation scope: three transfer checks under related signal-sensing conditions.

Dataset	Real Measurement?	Radar-Related?	Same Intra-Pulse Task?	CIL Protocol	Role in This Study
Measured comm.-I/Q (Real7) [34]	Yes	No (comm.)	Yes (mod. recog.)	3+2+2	Proxy: measured signal, different domain
NTIA compound radar [35,36]	Yes	Yes	No (waveform ID)	5+5+5+5	Proxy: real radar, different task
Radar-PRI [37]	Public	Yes	No (PRI recog.)	2+1+1+1	Proxy: radar domain, different modulation type

**Table 7:** Shared conditions and adaptation-specific differences. “-style” baselines are instantiated in the shared in-house comparison pipeline.

Item	Shared Setting Across All Methods
Backbone	Same dual-representation I/Q + STFT encoders (Section 3.2)
Replay memory	400 exemplars, class-balanced update across all sessions
Memory update rule	Same seeded random selection, class-balanced over seen classes
Training budget	Same epochs (base 5, inc. 3), same batch size (16)
Optimizer and scheduler	Adam; base learning rate $10^{-3}$ and weight decay $10^{-4}$
Data split and class order	Identical across all 12 seeds for all methods
Preprocessing	Same I/Q and time-frequency pipeline for all methods
Post-stage budget	MM-CILNet: 1 epoch, 400 examples, 25 steps, frozen encoders/gate, $lr=2\times 10^{-4}$
BiC-style adaptation	Distillation plus the common one-epoch balanced head update; no official held-out BiC layer
WA-style alignment	Post-hoc weight norm alignment per [7]

**Table 8:** Paired comparison between Replay and MM-CILNet on the main CIL-20 protocol at $S_{6}$ (12 matched seeds). Δ=MM-CILNet−Replay in percentage points; brackets are paired 95% confidence intervals, and *p* is the two-sided paired *t*-test value.

	Accuracy			
Metric	Replay	MM-CILNet	Δ [95% CI] (pp)	p-Value
All accuracy	0.5685±0.0215	0.5957±0.0230	+2.73[1.11,4.34]	0.0034
Old accuracy	0.5764±0.0227	0.6031±0.0258	+2.67[0.77,4.58]	0.0103
New accuracy	0.4974±0.0514	0.5295±0.0637	+3.21[−2.65,9.07]	0.2531
Avg. forgetting	0.1921±0.0484	0.1825±0.0244	−0.96[−4.00,2.08]	0.5004
Macro-F1	0.5885±0.0290	0.6157±0.0229	+2.73[1.04,4.41]	0.0045

**Table 9:** Comparison with representative class-incremental baselines on the final session $S_{6}$ of CIL-20. Results are reported over 12 random seeds as mean ± sample standard deviation. A dash marks an unavailable archived session-history aggregate.

Method	All Accuracy	Old Accuracy	New Accuracy	Avg. Forgetting
MM-CILNet	0.5952±0.0216	0.6034±0.0240	0.5217±0.0637	0.1826±0.0249
iCaRL-style	0.5608±0.0246	0.5612±0.0285	0.5564±0.0501	0.2270±0.0308
BiC-style	0.5445±0.0239	0.5385±0.0253	0.5990±0.0467	0.3336±0.0273
PODNet-style	0.5603±0.0237	0.5622±0.0312	0.5434±0.0688	0.2232±0.0312
DER-style	0.5226±0.0263	0.5245±0.0326	0.5052±0.0975	–
DER+head-FT-style	0.5621±0.0233	0.5693±0.0285	0.4974±0.0663	–
WA-style	0.5743±0.0162	0.5802±0.0140	0.5217±0.0568	–

**Table 10:** Equal-budget matched controls at $S_{6}$ (12 matched seeds; mean ± sample standard deviation). All enabled post stages process 400 examples in 25 steps. Bold indicates the best value in each metric column among the listed matched controls; higher is better for accuracy and lower is better for forgetting.

Fusion	Post Scope	Sampling	Tag	All	Old	New	Avg. Forgetting
Gate	None	–	G–N	0.5770±0.0183	0.5891±0.0157	0.4679±0.0976	0.1636±0.0436
Gate	Heads	Balanced	G–HB	0.5920±0.0226	0.6018±0.0264	0.5043±0.1014	0.1800±0.0274
Gate	Heads	Current+replay	G–HN	0.5874±0.0196	0.5948±0.0211	0.5208±0.0974	0.2178±0.0303
Gate	Full	Balanced	G–FB	0.6005±0.0217	0.6212±0.0241	0.4141±0.0948	0.1133±0.0356
Gate	Full	Current+replay	G–FN	0.6059±0.0139	0.6075±0.0185	0.5911±0.0635	0.2140±0.0223
Mean	None	–	M–N	0.5734±0.0126	0.5878±0.0152	0.4444±0.1238	0.2036±0.0266
Mean	Heads	Balanced	M–HB	0.5918±0.0183	0.6066±0.0255	0.4592±0.0771	0.1786±0.0297
I/Q only	None	–	W–N	0.5489±0.0244	0.5571±0.0255	0.4748±0.0960	0.1938±0.0274
I/Q only	Heads	Balanced	W–HB	0.5676±0.0264	0.5753±0.0291	0.4983±0.0601	0.1904±0.0309
STFT only	None	–	T–N	0.4990±0.0219	0.4999±0.0242	0.4905±0.0732	0.2659±0.0263
STFT only	Heads	Balanced	T–HB	0.5193±0.0217	0.5267±0.0253	0.4523±0.0626	0.2040±0.0362

**Table 11:** Head-only minus no-post-stage paired differences at $S_{6}$. Differences and paired 95% confidence intervals are in percentage points. Positive accuracy values favor the head-only stage; negative forgetting values indicate less forgetting. The *p*-values are unadjusted direct tests; multiplicity-controlled conclusions use the prespecified broader reviewer-control family.

Representation	Endpoint	Δ (pp)	95% CI (pp)	*p*
Gate	All	+1.50	[−0.26,3.26]	0.0872
Gate	Old	+1.26	[−0.72,3.25]	0.1882
Gate	New	+3.65	[−5.09,12.38]	0.3779
Gate	Forgetting	+1.65	[−1.90,5.19]	0.3288
Mean	All	+1.84	[0.36,3.32]	0.0195
Mean	Old	+1.88	[0.40,3.36]	0.0175
Mean	New	+1.48	[−7.76,10.71]	0.7318
Mean	Forgetting	−2.50	[−5.00,0.00]	0.0501
I/Q only	All	+1.88	[0.68,3.07]	0.0053
I/Q only	Old	+1.82	[0.87,2.77]	0.0014
I/Q only	New	+2.34	[−2.05,6.74]	0.2649
I/Q only	Forgetting	−0.34	[−1.77,1.09]	0.6107
STFT only	All	+2.03	[0.67,3.39]	0.0072
STFT only	Old	+2.68	[1.05,4.32]	0.0041
STFT only	New	−3.82	[−8.52,0.88]	0.1012
STFT only	Forgetting	−6.19	[−8.88,−3.50]	0.0004

**Table 12:** Direct Gate-versus-Mean comparison at $S_{6}$ (12 matched seeds). Values are All accuracy; *p* is from the paired *t*-test.

Evaluation Environment	Mean + Head FT	Gate + Head FT	*p*
AWGN	0.5918	0.5920	0.9805
Fading	0.3008	0.2987	0.7838
Fading + impulsive noise	0.0966	0.0973	0.8859

**Table 13:** Descriptive per-family accuracy at session $S_{6}$, pooled over 12 matched seeds. Δ denotes MM-CILNet minus Replay; inference is performed at the whole-run seed level in the matched controls.

Family	Replay	MM-CILNet	Δ
Basic (NS, LFM, Barker)	0.6493	0.7037	+0.0544
Phase (Frank, P1–P4)	0.4951	0.5031	+0.0080
Time/Freq. (Costas, T1–T4, NLFM, HFM, SFM)	0.6395	0.6582	+0.0187
Compound (BPSK-LFM, Frank-LFM, P4-LFM, SF-PC)	0.4575	0.5056	+0.0482
All (20 classes)	0.5685	0.5957	+0.0273

**Table 14:** Old/new class bias gap on the main CIL-20 protocol. A smaller value indicates more balanced predictions between old and new classes.

Method	Bias Gap	Reduction	*p*-Value
Replay	0.1887	–	–
MM-CILNet	0.1535	18.7%	0.0297

**Table 15:** SNR-stratified absolute All accuracy at $S_{6}$ (12 matched seeds; mean ± sample standard deviation). Both methods use the same class order, memory budget, and evaluated samples.

SNR (dB)	Replay	MM-CILNet
−18	0.0653±0.0194	0.0708±0.0267
−16	0.1097±0.0366	0.1333±0.0355
−14	0.1611±0.0385	0.2069±0.0609
−12	0.2778±0.0462	0.3319±0.0337
−10	0.3861±0.0714	0.3972±0.0602
−8	0.4681±0.0524	0.5139±0.0745
−6	0.5583±0.0712	0.6181±0.0665
−4	0.6931±0.0668	0.7347±0.0539
−2	0.7681±0.0515	0.8000±0.0508
0	0.7639±0.0431	0.7861±0.0283
+2	0.8264±0.0479	0.8458±0.0370
+4	0.7972±0.0512	0.8125±0.0294
+6	0.8181±0.0351	0.8403±0.0337
+8	0.7903±0.0392	0.8097±0.0429
+10	0.8111±0.0372	0.8167±0.0492
+12	0.8014±0.0417	0.8139±0.0354

**Table 16:** Measured communication-I/Q validation (Real7, 3+2+2 protocol, 17 seeds).

Method	All Accuracy	Old Accuracy	New Accuracy	Avg. Forgetting
MM-CILNet	0.7585±0.0114	0.6815±0.0154	0.9511±0.0090	0.1698±0.0207
PODNet-style	0.7368±0.0283	0.6505±0.0409	0.9526±0.0068	0.2053±0.0463
iCaRL-style	0.7253±0.0344	0.6346±0.0485	0.9520±0.0077	0.2246±0.0587
BiC-style	0.6869±0.0287	0.5784±0.0401	0.9580±0.0006	0.2980±0.0489

**Table 17:** NTIA P0N compound radar waveforms (5+5+5+5 protocol, 11 seeds).

Method	All Accuracy	Old Accuracy	New Accuracy	Avg. Forgetting
MM-CILNet	0.6043±0.0277	0.5379±0.0353	0.9366±0.0289	0.3634±0.0523
PODNet-style	0.5054±0.0213	0.4339±0.0200	0.8627±0.0563	0.4490±0.0268
iCaRL-style	0.5006±0.0311	0.4284±0.0303	0.8617±0.0691	0.4305±0.0595
BiC-style	0.4882±0.0275	0.4188±0.0330	0.8352±0.0593	0.5290±0.0333

**Table 18:** Radar-PRI transfer validation (2+1+1+1 protocol, 24 seeds).

Method	All Accuracy	Old Accuracy	New Accuracy	Avg. Forgetting
MM-CILNet	0.8301±0.1014	0.8770±0.0887	0.6333±0.4706	0.0475±0.0849
BiC-style	0.7276±0.1063	0.8095±0.1041	0.3833±0.4752	0.0255±0.0463
PODNet-style	0.6635±0.1010	0.7718±0.0910	0.2083±0.3889	0.0127±0.0183
iCaRL-style	0.6522±0.1030	0.7560±0.0956	0.2167±0.4125	0.0197±0.0289

**Table 19:** Descriptive Old-accuracy difference between MM-CILNet and the strongest listed adaptation. Different tasks and implementations prevent mechanism-level aggregation.

Setting	Strongest Baseline	MM-CILNet Old	ΔOld (pp)
CIL-20 (8+2×6)	WA-style (0.5802)	0.6034	+2.32
Real7 meas. I/Q (3+2+2)	PODNet (0.6505)	0.6815	+3.10
NTIA compound (5+5+5+5)	PODNet (0.4339)	0.5379	+10.40
Radar-PRI (2+1+1+1)	BiC-style (0.8095)	0.8770	+6.75

**Table 20:** Matched external Replay-only versus Replay-plus-head-fine-tuning checks. Δ is the paired final-session difference in percentage points.

Dataset	Seeds	ΔAll	$p_{\mathrm{All}}$	ΔOld	$p_{\mathrm{Old}}$
Real7	17	+1.57	0.0216	+2.25	0.0205
NTIA P0N	11	+1.17	0.3443	+1.12	0.4564
Radar-PRI	17	+3.85	0.1643	+1.68	0.5135

**Table 21:** Memory-budget sensitivity of the complete post-stage protocol at $S_{6}$ (12 matched seeds per budget). ΔOld=MM-CILNetOld−ReplayOld. The 400-sample row is the primary cohort; comparisons are paired within each budget.

Budget	Replay All	Replay Old	MM-CILNet All	MM-CILNet Old	ΔOld (pp)
200	0.5339	0.5338	0.5345	0.5344	+0.07
400	0.5685	0.5764	0.5957	0.6031	+2.67
800	0.6253	0.6423	0.6445	0.6609	+1.86

**Table 22:** Class-order sensitivity at $S_{6}$ (12 seeds, 5 random orders). ΔOld computed against the Replay baseline under the same order.

Order	Replay Old	MM-CILNet Old	ΔOld (pp)
Order 1 (s = 2026)	0.5273	0.5436	+1.63
Order 2 (s = 42)	0.5484	0.5676	+1.92
Order 3 (s = 123)	0.5541	0.5778	+2.37
Order 4 (s = 777)	0.5108	0.5422	+3.14
Order 5 (s = 999)	0.5554	0.5782	+2.29
Mean	0.5392	0.5619	+2.27

**Table 23:** Replay-allocation stress test at $S_{6}$ (12 matched seeds; mean ± sample standard deviation). Recent-heavy uses a 2.5:1 per-class allocation weight for current versus old classes, a 400-sample memory, and no replacement. Lower forgetting is better.

Memory Allocation	Post Stage	All	Old	New	Avg. Forgetting
Balanced	None	0.5770±0.0183	0.5891±0.0157	0.4679±0.0976	0.1636±0.0436
Balanced	Heads	0.5920±0.0226	0.6018±0.0264	0.5043±0.1014	0.1800±0.0274
Recent-heavy	None	0.5634±0.0125	0.5728±0.0134	0.4783±0.1516	0.2105±0.0221
Recent-heavy	Heads	0.5748±0.0204	0.5817±0.0181	0.5130±0.1506	0.2167±0.0262

## Data Availability

An anonymized reproducibility package accompanies the manuscript and contains the CIL-20 generator, fixed protocol and metadata files, training and baseline adaptations, matched-control configurations, seed-level metrics, experimental configurations and sampling records, and the statistical analysis script. The generated waveform arrays are available from the corresponding author because of repository size limits. The reproducibility package is provided as Supplementary Materials. The public measured communication-I/Q dataset is available from Mendeley Data at https://doi.org/10.17632/tjzsbph49x.2 (accessed on 25 May 2026). The NTIA compound radar waveforms are available from https://catalog.data.gov/dataset/compound-radar-waveforms-data (accessed on 25 May 2026). The Radar-PRI dataset is available from Mendeley Data at https://doi.org/10.17632/skjvm46zn7.1 (accessed on 25 May 2026).

## Supplementary Materials

The following supporting information can be downloaded at: https://www.mdpi.com/article/10.3390/s26175494/s1.
